# Optogenetic Evidence for the Intrinsic Phase Separation Propensity of the Sgs1 N-Terminal Region: Implications for Assemblysome Formation

**DOI:** 10.3390/biom16091240

**Published:** 2026-08-27

**Authors:** Bence György Gombás, Erika Gábor, Viktor Honti, Orsolya Németh-Szatmári, Ferenc Jankovics, Zoltán Villányi

**Affiliations:** 1 Department of Biochemistry and Molecular Biology, Faculty of Science and Informatics, University of Szeged, 6726 Szeged, Hungary; gombasbencegyorgy97@gmail.com (B.G.G.); orsolyaszatmari7@gmail.com (O.N.-S.); 2 Drosophila Blood Cell Differentiation Group, Institute of Genetics, HUN-REN Biological Research Centre, 6726 Szeged, Hungary; gabor.erika@brc.hu (E.G.); honti.viktor@brc.hu (V.H.); 3 Laboratory of Drosophila Germ Cell Differentiation, Institute of Genetics, HUN-REN Biological Research Centre, 6726 Szeged, Hungary; jankovic@brc.hu; 4 Department of Medical Biology, Albert Szent-Györgyi Medical Centre, University of Szeged, 6720 Szeged, Hungary

**Keywords:** assemblysome, liquid–liquid phase separation, Sgs1, optoDroplet, ribosome-nascent chain complex, biomolecular condensates

## Abstract

Assemblysomes are ribosome-nascent chain condensates that regulate co-translational processes through liquid–liquid phase separation, yet the sequence determinants underlying their formation remain incompletely understood. Previous studies identified the DNA helicase Sgs1 as an assemblysome-associated protein; however, whether its N-terminal region possesses intrinsic phase separation propensity has not been experimentally examined. Here, we investigated the first 135 amino acids of Sgs1 using a light-inducible optoDroplet assay. A mCherry–Cry2–Sgs1_1–135_ fusion construct was compared with the established positive control FUS–mCherry–Cry2 and the negative control mCherry–Cry2 in live HEK293T cells. Following blue-light activation, Sgs1_1–135_ reproducibly formed reversible condensates, indicating intrinsic phase separation propensity. Quantitative image analysis revealed light-dependent increases in condensate number, average condensate area, and integrated condensate fluorescence intensity. Compared with FUS, Sgs1_1–135_ formed slightly fewer and smaller condensates but displayed reproducible light-dependent condensate formation. These findings indicate that the Sgs1 N-terminal region exhibits intrinsic phase separation propensity in a validated optogenetic assay. Although this proof-of-principle study does not establish the molecular mechanism of assemblysome formation, the results are consistent with the hypothesis that the Sgs1 N-terminus may contribute to the multivalent interactions underlying assemblysome organization.

## 1. Introduction

Cells frequently need to delay protein synthesis while retaining the ability to rapidly complete translation when environmental conditions change. Assemblysomes have recently emerged as ribosome-nascent chain condensates (RNCs) that may provide such a regulatory mechanism through liquid–liquid phase separation (LLPS) [1]. Membraneless organelles formed through LLPS have emerged as fundamental regulators of intracellular organization by creating dynamic biochemical compartments without membrane boundaries [2]. These condensates participate in diverse cellular processes, including RNA metabolism, protein homeostasis, co-translational assembly, signal transduction, and stress adaptation [1,3]. Although the molecular principles governing many biomolecular condensates have been extensively investigated, the mechanisms underlying the formation of ribosome-associated condensates remain incompletely understood [4].

Unlike stress granules or P-bodies, assemblysomes contain actively translating ribosomes stalled during elongation and remain resistant to EDTA and cycloheximide treatment, reflecting their distinct molecular organization [1]. Current evidence suggests that assemblysomes function as transient reservoirs of partially synthesized proteins, allowing rapid completion of translation when cells require an immediate increase in protein production, such as during proteotoxic or genotoxic stress [1,5,6,7].

A central question in assemblysome biology is how these condensates are initiated. Current models propose that LLPS is driven by multivalent interactions involving nascent polypeptide chains emerging from stalled ribosomes together with RNA and assembly-associated proteins. Supporting this model, the intrinsically disordered N-terminal region of the proteasome subunit Rpt1 was previously shown to promote condensate formation, suggesting that sequence elements exposed early during translation contribute to assemblysome formation [6,7]. Whether this principle extends to other assemblysome-associated proteins, however, remains unknown.

The RecQ-family DNA helicase Sgs1 is an attractive candidate for addressing this question [8]. Sgs1 plays a central role in homologous recombination and DNA double-strand break repair and has previously been identified as an assemblysome-associated protein [6,9,10]. Ribosome-nascent chain complexes containing Sgs1 accumulate in assemblysomes under basal conditions and undergo stress-dependent redistribution to translating ribosomes following DNA damage [6]. While these observations establish a functional association between Sgs1 and assemblysomes, they do not determine whether the Sgs1 polypeptide itself possesses intrinsic condensate-promoting activity or whether condensate formation primarily depends on the native assemblysome environment, including ribosomes, RNA, and associated proteins.

To distinguish between these possibilities, we employed the optoDroplet system, a well-established optogenetic assay in which light-induced Cry2 oligomerization promotes local enrichment of the fusion proteins, allowing their concentration to exceed the saturation threshold required for phase separation [11,12,13,14]. Rather than reproducing the native molecular architecture of assemblysomes, this experimental system provides a standardized framework for assessing whether an individual protein sequence possesses intrinsic phase separation propensity in living cells. By minimizing the contribution of additional assemblysome components, the assay enables direct comparison of candidate sequences under identical experimental conditions.

Here, we investigated the first 135 amino acids of Sgs1, a predicted intrinsically disordered N-terminal region that emerges early from the ribosome during translation [6]. Using the optoDroplet assay, we asked whether this region alone is capable of promoting condensate formation [11]. We found that Sgs1_1–135_ reproducibly formed reversible, light-induced condensates in living cells, demonstrating that this region possesses intrinsic phase separation propensity in this experimental system. Although this proof-of-principle study does not recapitulate the full molecular complexity of native assemblysomes, our findings are consistent with the hypothesis that the Sgs1 N-terminal region may contribute to condensate formation as one component of the multivalent interaction network underlying assemblysome organization. This experimental design allowed us to isolate the contribution of the Sgs1 N-terminal sequence from the additional molecular interactions present in native assemblysomes, thereby providing a controlled assessment of its intrinsic phase separation propensity.

## 2. Materials and Methods

### 2.1. Plasmid Construction

The DNA sequence encoding amino acids 1–135 of the *Saccharomyces cerevisiae SGS1* open reading frame was amplified by PCR using gene-specific primers (Table 1). The amplified fragment was cloned into a mammalian expression vector encoding mCherry and the photolyase homology region of *Arabidopsis thaliana* Cryptochrome 2 (Cry2) using the NEBuilder^®^ HiFi DNA Assembly Kit (New England Biolabs, Ipswich, MA, USA) according to the manufacturer’s instructions. The resulting construct expressed the fusion protein mCherry–Cry2–Sgs1_1–135_ from an SFFV constitutive promoter.

The previously validated FUS–mCherry–Cry2 construct was used as a positive control for optogenetically induced condensate formation, whereas mCherry–Cry2 served as the negative control [11]. The FUS construct was included to verify the performance of the optoDroplet assay rather than to model the structural organization of Sgs1. All plasmid constructs were verified by Sanger sequencing before use.

### 2.2. Cell Culture and Transient Transfection

HEK293T cells (ATCC CRL-3216, Manassas, VA, USA) were maintained in Dulbecco’s Modified Eagle Medium (Capricorn Scientific, DMEM-HPA, Ebsdorfergrund, Germany) supplemented with 10% heat-inactivated fetal bovine serum (Capricorn Scientific, 10-FBS-HI-12F, Ebsdorfergrund, Germany) and 1% penicillin–streptomycin (Capricorn Scientific, PS-B, Ebsdorfergrund, Germany) at 37 °C in a humidified incubator containing 5% CO_2_.

For live-cell imaging, 10^4^ cells were seeded into eight-well glass-bottom μ-Slides (Ibidi, 80827, Gräfelfing, Germany) approximately 24 h before transfection to achieve 60–80% confluence at the time of DNA delivery. Cells were transiently transfected with optoDroplet plasmids using jetOPTIMUS^®^ (Sartorius, 101000051, Göttingen, Germany) following the manufacturer’s protocol. Identical amounts of plasmid DNA were used for each construct.

Immediately before imaging, the culture medium was replaced with Live Cell Imaging Solution (Invitrogen, A14291DJ, Eugene, OR, USA) to maintain physiological conditions throughout microscopy.

### 2.3. Optogenetic Phase Separation Assay

The intrinsic phase separation propensity of the Sgs1 N-terminal region was evaluated using the optoDroplet system, in which blue-light-induced Cry2 oligomerization increases the local concentration of the fused protein sequence, thereby promoting condensate formation when the phase separation threshold is reached [11].

Blue-light activation was performed using repeated 488 nm laser illumination (600 ms exposure at 1.988% laser power), while fluorescence images were acquired continuously before, during, and after illumination to monitor condensate formation and reversibility.

### 2.4. Live-Cell Imaging

Live-cell imaging was performed using a VisiScope spinning-disk confocal microscope (Visitron Systems, Puchheim, Germany) equipped with a Yokogawa CSU-W1 scanning unit (Musashino, Japan) and Andor Zyla 4.2 PLUS sCMOS cameras (Belfast, Northern Ireland, UK). Images were acquired using a PlanApo N 60× oil-immersion objective 1.42 NA (Olympus, Hachioji, Japan).

For all experimental groups, identical microscope settings, laser power, exposure times, and image acquisition parameters were maintained throughout this study. Time-lapse image series were collected immediately before blue-light activation and continued throughout the activation period to capture the kinetics of condensate formation.

Representative images shown in the figures were selected from experiments performed under identical acquisition conditions and are representative of observations obtained in independent biological replicates.

### 2.5. Image Analysis

Quantitative image analysis was performed using Fiji/ImageJ (version: 1.54p) (National Institutes of Health, Madison, WI, USA). Condensates were identified using a standardized image-processing workflow that was applied identically to all experimental groups. Image segmentation parameters and intensity thresholds were kept constant throughout the analysis to minimize user-dependent bias.

For each cell, three quantitative parameters were determined: (3a) the number of condensates per cell, (3b) the integrated condensate fluorescence intensity, and (3c) the mean condensate area. Integrated condensate fluorescence intensity was calculated as the sum of the integrated density values of all condensates within each cell. These parameters were selected to characterize condensate formation, growth, and fluorescence accumulation following optogenetic activation.

To reduce potential bias arising from differences in transfection efficiency, quantitative analyses were performed on cells exhibiting comparable fluorescence intensity and normal cellular morphology.

### 2.6. Statistical Analysis

Quantitative analyses were performed using data obtained from two independent biological experiments, with at least 11 cells analyzed per construct. Individual cells were treated as technical observations within each biological replicate.

Data are presented as mean ± SEM. Statistical significance between experimental groups at corresponding time points was evaluated using an unpaired two-tailed Student’s *t*-test. Significant differences are indicated (*) in the corresponding figures. Differences were considered statistically significant at *p* < 0.05.

Graphical representation, statistical analyses and visualization were performed using Microsoft Excel and PowerPoint (Microsoft Corporation, Redmond, WA, USA), Fiji/ImageJ (National Institutes of Health, Madison, WI, USA) and BioRender (https://BioRender.com).

## 3. Results

### 3.1. Experimental Design

**Figure 1:**
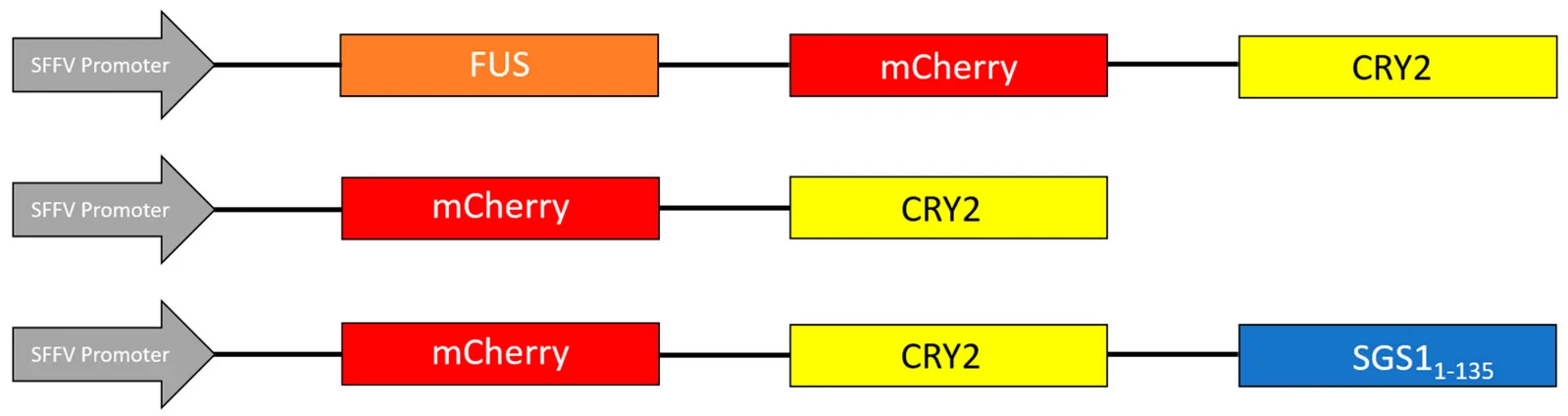
Optogenetic constructs used in this study. Schematic representation of FUS–mCherry–Cry2, mCherry–Cry2, and mCherry–Cry2–Sgs1_1–135_ constructs used to assess phase separation.

To investigate whether the N-terminal region of Sgs1 possesses intrinsic condensate-promoting activity, we generated a light-inducible fusion protein containing amino acids 1–135 of *Saccharomyces cerevisiae* Sgs1 fused to mCherry and Cry2 (Figure 1). The construct was compared with the established optoDroplet positive control FUS–mCherry–Cry2 and the negative control mCherry–Cry2 under identical experimental conditions. Since our aim was to determine whether the N-terminal region of Sgs1 is capable of inducing LLPS on its own, comparison with the negative control alone would have been sufficient to address the objective of the experiment. The extensively validated positive control used in this experiment was included to confirm that the optoDroplet assay was performed correctly, rather than to serve as a direct biological comparison.

Blue-light illumination induced Cry2 oligomerization, allowing condensate formation to be monitored in living HEK293T cells by time-lapse confocal microscopy.

### 3.2. FUS Validates the optoDroplet Assay

**Figure 2:**
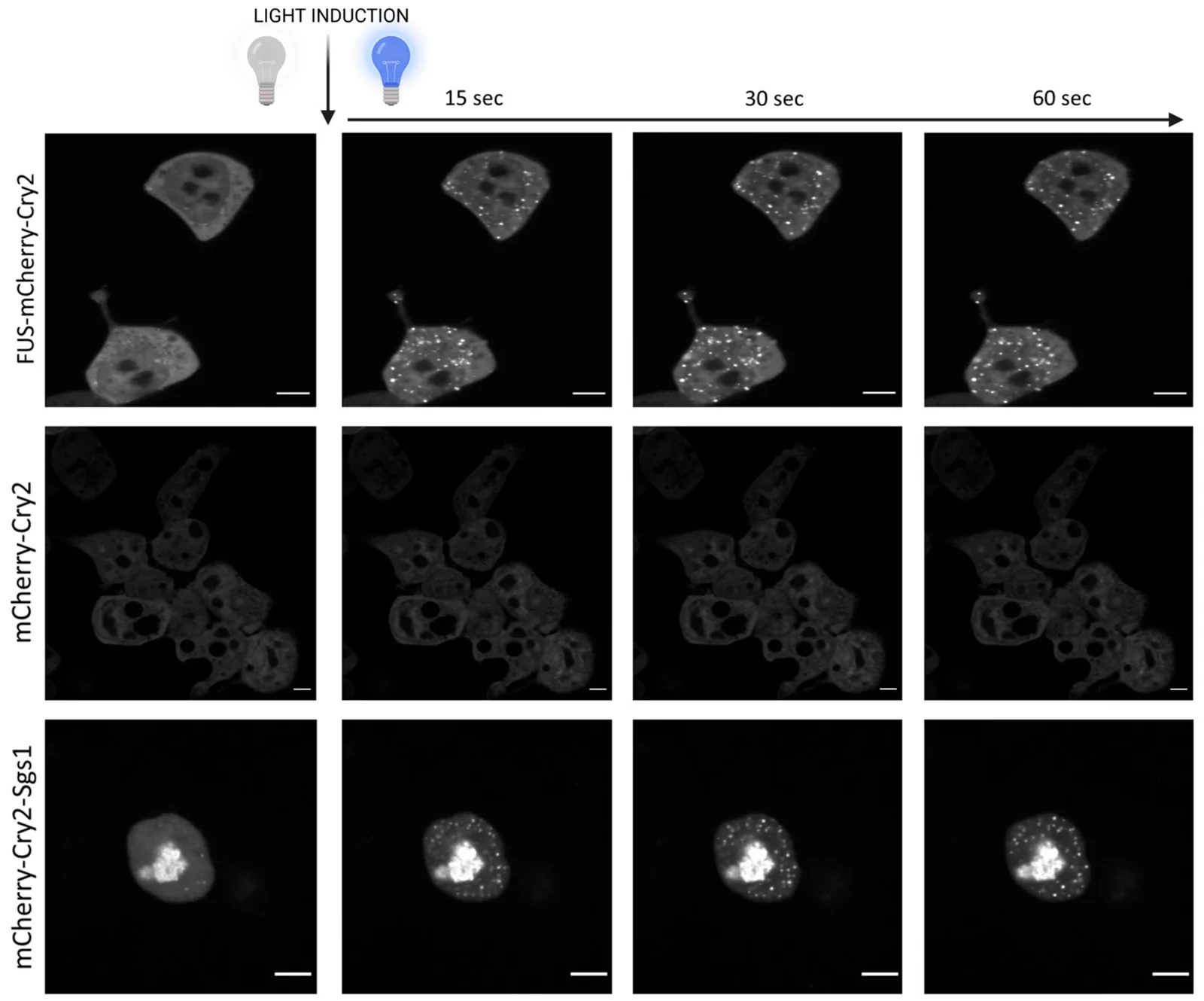
Light-induced condensate formation. Representative images showing blue-light-induced condensate formation in cells expressing the indicated constructs. FUS and Sgs1_1–135_ form condensates, while mCherry–Cry2 remains diffuse. Scale bars: 5 µm. Created in BioRender. Gombás, B. (2026) https://BioRender.com/43xr469 (access on 21 August 2026).

Illumination of cells expressing FUS–mCherry–Cry2 resulted in the rapid appearance of numerous intracellular condensates (Figure 2). Condensates increased in number and size during the activation period and gradually disappeared after termination of blue-light-triggered initiation of condensate formation, consistent with the reversible behavior expected for the optoDroplet system.

In contrast, cells expressing mCherry–Cry2 alone remained largely diffuse throughout the experiment and showed no comparable condensate formation.

These observations confirmed the expected performance of the optogenetic assay and established an experimental reference for comparison with the Sgs1 construct.

### 3.3. Sgs1_1–135_ Forms Light-Dependent Condensates

Cells expressing mCherry–Cry2–Sgs1_1–135_ also formed condensates following blue-light activation (Figure 2). Condensates appeared rapidly after illumination and remained detectable throughout the imaging period. Following cessation of illumination, condensates gradually dispersed, indicating that condensate formation was reversible under the experimental conditions used. The enlarged nuclear fluorescent structure observed in the representative image most likely reflects basal accumulation of the expressed fusion protein in mCherry–Cry2–Sgs1_1–135_-transfected cells rather than optogenetically induced condensate formation. The molecular basis of this nuclear enrichment was not investigated in the present study.

No comparable condensate formation was observed in the negative control.

Together, these observations demonstrate that the Sgs1 N-terminal construct exhibits reproducible light-dependent condensate formation in the optoDroplet assay.

### 3.4. Quantitative Analysis of Condensate Formation

**Figure 3:**
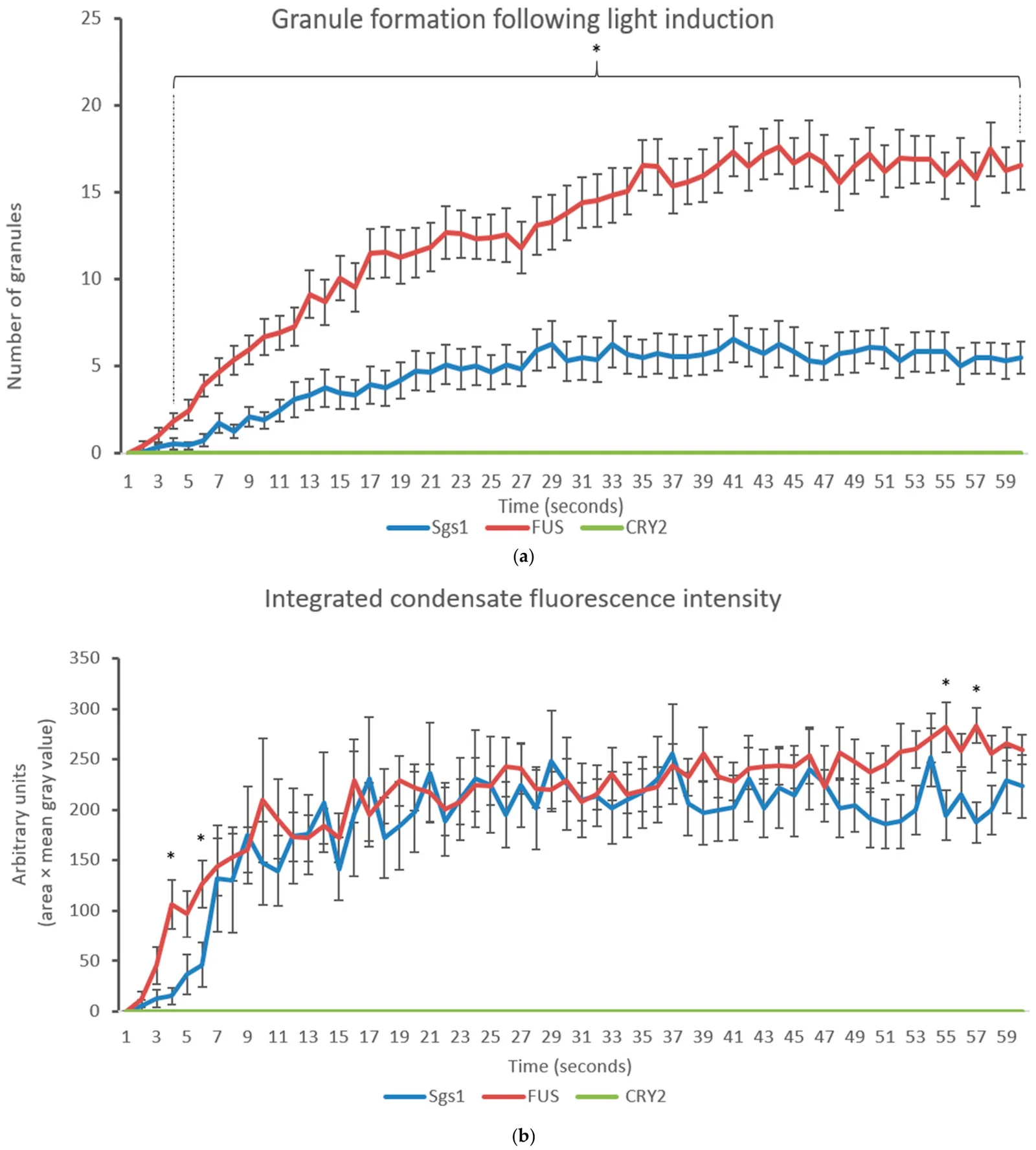

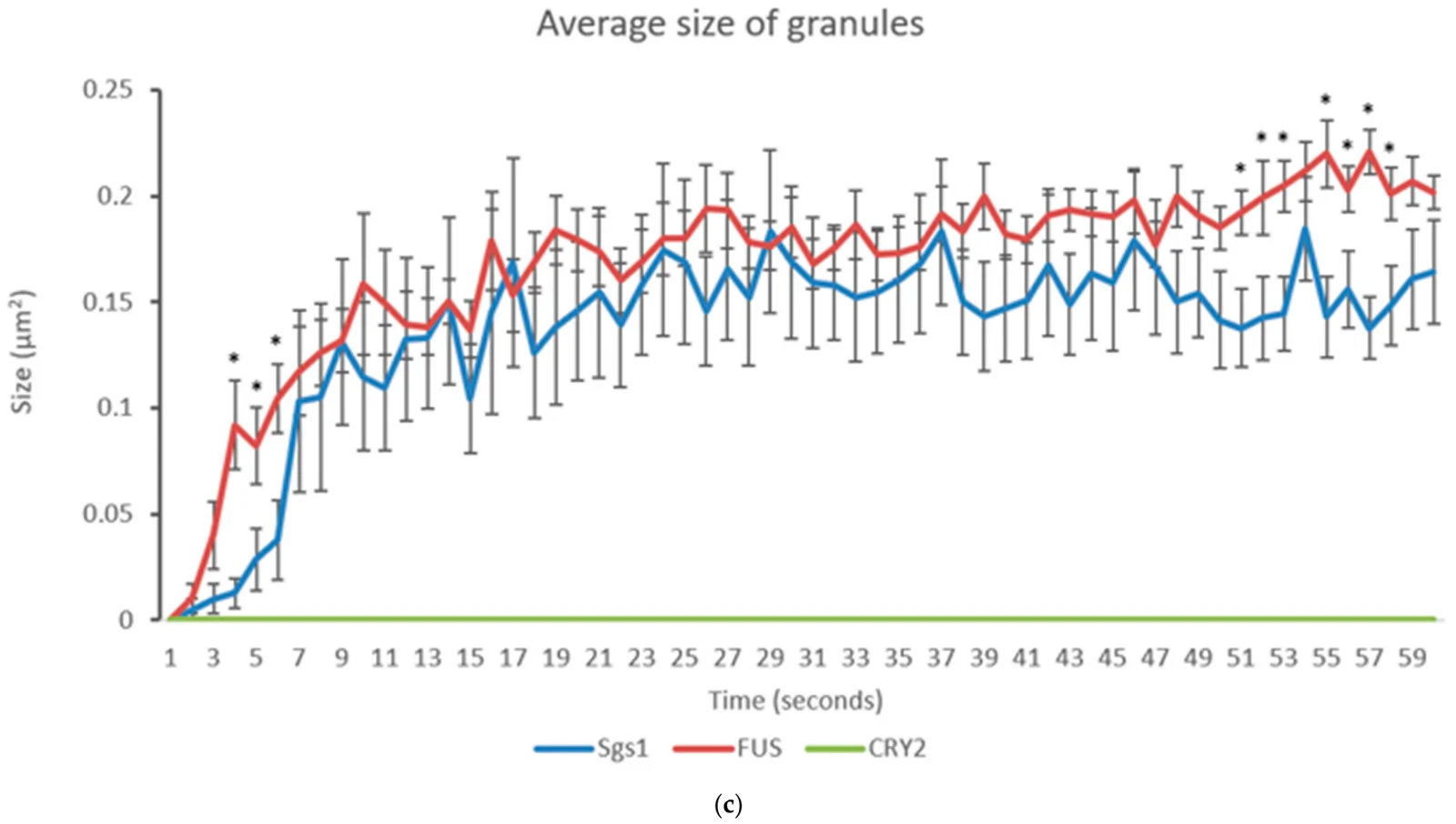
Quantitative analysis of condensate formation. Blue-light-induced changes in granule number, integrated condensate fluorescence intensity, and average granule size over time. Statistical significance is determined by two-tailed Student’s *t*-test (* *p* < 0.05). (**a**) Granule number per cell over time; (**b**) integrated condensate fluorescence intensity per cell over time; (**c**) average granule size per cell over time. Sgs1: mCherry–Cry2–Sgs1_1–135_; FUS: FUS–mCherry–Cry2; CRY2: mCherry–Cry2 negative control.

Quantitative image analysis was performed by measuring the condensate number, average condensate area, and integrated condensate fluorescence intensity (area × mean gray value) per cell throughout the imaging period. The analysis focused on the dynamic appearance of light-induced condensates during time-lapse imaging rather than on static fluorescence patterns observed before activation (Figure 3).

FUS generated a significantly greater number of condensates than Sgs1_1–135_ at multiple time points following blue-light activation (Figure 3a). Nevertheless, both constructs displayed a rapid increase in condensate number immediately after illumination, followed by stabilization during the later stages of imaging.

Integrated condensate fluorescence intensity increased following activation in both constructs (Figure 3b). Although FUS generally exhibited higher fluorescence values, differences between the two constructs were smaller than those observed for condensate number and did not reach statistical significance at every time point.

Similarly, the average condensate area increased rapidly after illumination for both constructs (Figure 3c). Condensates formed by Sgs1_1–135_ remained smaller on average than those formed by FUS throughout most of the observation period.

Overall, both constructs exhibited reproducible light-dependent condensate formation, although the extent of condensate formation differed quantitatively under the experimental conditions used.

## 4. Discussion

Biomolecular condensates formed through LLPS have emerged as a fundamental mechanism for intracellular organization [15,16,17,18]. The objective of this study was to determine whether the N-terminal region of Sgs1 possesses intrinsic phase separation propensity using a standardized optoDroplet assay. Accordingly, we evaluated the Sgs1_1–135_ construct together with established negative and positive controls under identical experimental conditions. These controls serve distinct and complementary purposes: mCherry–Cry2 provides the relevant negative control for determining whether addition of Sgs1_1–135_ confers light-dependent condensate-forming propensity, whereas FUS–mCherry–Cry2 serves as a validated positive control confirming the performance of the OptoDroplet assay. FUS was therefore not intended as a sequence-, size-, charge-, or topology-matched biological comparator for Sgs1_1–135_. Because the optoDroplet system sensitively measures the condensate-forming propensity of individual protein sequences, it was used to evaluate the intrinsic behavior of the Sgs1 N-terminus rather than to reproduce the molecular architecture of native assemblysomes.

Assemblysomes were first described as Not1-containing ribosome-nascent chain assemblies involved in co-translational proteasome assembly, particularly for Rpt1 and Rpt2, where ribosome pausing enables nascent-chain interactions during protein complex formation [7]. Although numerous assemblysome-associated proteins have been identified, the sequence features underlying their condensate-forming properties remain poorly understood.

Our experiments show that Sgs1_1–135_ forms reversible, light-induced condensates in the optoDroplet assay. Whereas the negative control (mCherry–Cry2) remained diffuse, the positive control (FUS–mCherry–Cry2) exhibited the expected condensate formation. Quantitative analysis further demonstrated light-dependent increases in condensate number, area and integrated fluorescence intensity. FUS is a canonical low-complexity, IDR-rich phase-separating protein, and its N-terminal region has been widely used as a strong LLPS driver [11,19,20,21]. Compared with FUS, Sgs1_1–135_ formed fewer and smaller condensates, whereas differences in integrated fluorescence were less pronounced. Together, these results indicate that the Sgs1 N-terminal region possesses intrinsic condensate-forming propensity while displaying condensate dynamics distinct from the canonical FUS construct.

These findings should be interpreted within the scope of the experimental model. The optoDroplet assay measures the intrinsic condensate-forming propensity of isolated protein sequences in living cells but does not reproduce the molecular composition of native assemblysomes. Consequently, our experiments neither establish the mechanism of assemblysome formation nor define the physiological contribution of Sgs1 to the emergence of endogenous ribosome-nascent chain condensates. Rather, they demonstrate that the isolated Sgs1 N-terminus is sufficient to support condensate formation in a standardized optogenetic system.

Nevertheless, our results complement previous observations identifying Sgs1 as an assemblysome-associated protein. Sgs1, the yeast ortholog of BLM, functions together with Top3 and Rmi1 in homologous recombination and DNA double-strand break repair, including DNA end resection and dissolution of recombination intermediates [9,22,23,24,25,26]. Because inappropriate helicase activity threatens genome stability, storage of Sgs1 as a paused ribosome-nascent chain complex could provide an efficient regulatory mechanism, allowing rapid completion of translation following genotoxic stress [8,23,27]. Consistent with this model, SGS1 mRNA relocates from assemblysomes to translating polysomes after DNA damage [6]. Our data extend these observations by showing that the Sgs1 N-terminal region itself possesses intrinsic condensate-forming propensity, supporting the hypothesis that it contributes to assemblysome formation under native conditions.

An additional implication concerns the position of the condensate-forming sequence. Because the intrinsically disordered N-terminal region emerges first from the ribosomal exit tunnel, it becomes available for intermolecular interactions before synthesis of the full-length protein is complete. Intrinsically disordered regions function as flexible interaction platforms whose sequence-encoded multivalency promotes numerous weak, transient interactions that collectively drive biomolecular condensate formation [28,29,30,31]. Positioning such a region at the N-terminus may therefore represent an evolutionarily advantageous strategy, enabling early interactions with neighboring nascent chains, RNA or assembly-associated proteins while translation is still in progress [32,33,34]. Although this mechanism was not directly tested here, our observations are consistent with the idea that early-emerging intrinsically disordered segments contribute to co-translational assemblysome organization.

More broadly, the timing of sequence emergence during translation may itself represent an additional layer of condensate regulation. Rather than serving solely as passive structural elements, N-terminal intrinsically disordered regions may transiently increase local molecular connectivity during protein synthesis. Such a mechanism is particularly attractive for assemblysomes, whose formation depends on partially synthesized nascent chains remaining associated with translating ribosomes [6,7]. Optogenetic phase separation assays therefore provide a complementary approach for dissecting sequence-intrinsic condensate-forming properties while separating them from the complexity of the native assemblysome environment [3,11,30].

FUS was included exclusively as a validated positive control for the optoDroplet assay rather than as a biological equivalent of Sgs1. Accordingly, quantitative differences between FUS and Sgs1_1–135_ should not be interpreted as differences in biological “strength” but simply demonstrate that the assay discriminates between proteins with distinct condensate-forming behaviors under identical experimental conditions.

Several limitations should be acknowledged. Only the N-terminal 135 amino acids of Sgs1 were examined, and the assay does not capture interactions involving the remainder of the protein, stalled ribosomes, RNA or other assemblysome components. Nor does it address the influence of protein expression levels, endogenous interaction partners or native assemblysome organization. Future studies incorporating full-length Sgs1, alternative fusion architectures, endogenous expression systems and in vitro reconstitution will be required to define the physiological contribution of Sgs1 to assemblysome assembly.

In conclusion, the Sgs1 N-terminal region exhibits reproducible intrinsic phase separation propensity in a validated optogenetic assay. Although these experiments do not establish the molecular mechanism of assemblysome formation, they support the hypothesis that the Sgs1 N-terminus represents one sequence element capable of contributing to condensate formation within the multicomponent environment of native assemblysomes.

## 5. Conclusions

In this study, we investigated whether the N-terminal region of Sgs1 possesses intrinsic condensate-forming propensity. Using the optoDroplet assay, we demonstrated that Sgs1_1–135_ reproducibly forms reversible, light-induced condensates in HEK293T living cells. Comparison with the negative control was sufficient to demonstrate this activity, while the validated FUS positive control confirmed the proper performance of the assay. Together, these findings establish that the isolated Sgs1 N-terminal region is capable of promoting condensate formation under the experimental conditions used.

Importantly, this finding does not demonstrate that Sgs1_1–135_ alone is sufficient to form endogenous assemblysomes or define the molecular mechanism underlying their formation. Rather, it identifies the Sgs1 N-terminal region as a sequence element with intrinsic condensate-forming propensity that may contribute to the multivalent interactions underlying assemblysome organization. This interpretation is consistent with the position of the intrinsically disordered N-terminal region, which emerges early during translation and could therefore participate in interactions before synthesis of the full-length protein is completed.

Overall, our results provide experimental evidence that the Sgs1 N-terminus has intrinsic condensate-forming propensity and support its potential contribution to assemblysome organization. While these results do not provide a complete explanation of the molecular basis underlying assemblysome formation, they contribute to a better understanding of this process. Future studies using full-length Sgs1 and more physiologically relevant experimental systems will be required to determine whether and how this intrinsic activity contributes to the formation and function of endogenous assemblysomes.

## Figures and Tables

**Table 1:** Primers used to amplify DNA fragments for cloning.

Sgs1 forward primer	5′–CCGGGATCCACCGGTCGCCATGGTGACGAAGCCGTCACATAAC–3′
Sgs1 reverse primer	5′–ATCCGCTCCCTGAACCGGTACAAGAAAGCTGGGTC–3′
Plasmid forward primer 1	5′–ACCGGTTCAGGGAGCGGAT–3′
Plasmid reverse primer 1	5′–TCAGTTCCATAGGTTGGAAT–3′
Plasmid forward primer 2	5′–ATTCCAACCTATGGAACTGATGAAT–3′
Plasmid reverse primer 2	5′–CGACCGGTGGATCCCGGGCTGCTGCTCCGATCATGAT–3′

## Data Availability

The original contributions presented in this study are included in the article/Supplementary Materials. Further inquiries can be directed to the corresponding author.

## Supplementary Materials

The following supporting information can be downloaded at https://www.mdpi.com/article/10.3390/biom16091240/s1, Videos: Movie S1: Representative video of the optoDroplet assay in HEK293T cells transfected with the mCherry-Cry2 construct, used as a negative control, Movie S2: Representative video of the optoDroplet assay in HEK293T cells transfected with the FUS-mCherry-Cry2 construct, used as a positive control, Movie S3: Representative video of the optoDroplet assay in HEK293T cells transfected with the mCherry-Cry2-Sgs1_1–135_ construct. Table S1: Quantified raw data for condensates in cells expressing the different constructs.
